# Demoralization: Where it stands-and where we can take it: A bibliometric analysis

**DOI:** 10.3389/fpsyg.2022.1016601

**Published:** 2022-10-20

**Authors:** Qingyong Zheng, Lu Xiong, Huijun Li, Ming Liu, Jianguo Xu, Xiaofeng Luo

**Affiliations:** ^1^School of Public Health, Lanzhou University, Lanzhou, Gansu, China; ^2^Evidence-Based Nursing Center, School of Nursing, Lanzhou University, Lanzhou, Gansu, China; ^3^Evidence-Based Medicine Center, School of Basic Medical Sciences, Lanzhou University, Lanzhou, Gansu, China

**Keywords:** psychological distress, development history, COVID-19, bibliometrics, data visualization, demoralization

## Abstract

**Objectives:**

The purpose is to analyze existing studies related to the field of demoralization through bibliometrics.

**Methodology:**

Relevant literature on demoralization was searched from PubMed, Web of Science, the Cochrane Library, and CINAHL Complete. Bibliometric analysis was performed using GraphPad Prisma 8.2.1, VOSviewer 1.6.18 and R software. Research publication trends, author-country collaboration, research hotspots and future trends were explored by generating network relationship maps.

**Results:**

A total of 1,035 publications related to the field of demoralization were identified. The earliest relevant studies have been published since 1974, and the studies have grown faster since 2000. *Psyche-oncology* and Psychother Psychosom had the highest number of publications (*n* = 25). The United States, Italy and Australia have made outstanding contributions to the field and there was an active collaboration among leading scholars. Major research hotspots include the multiple ways of assessing demoralization, the specificity of various demographics and psychological disorders in different disease contexts, and the association and distinction of diverse clinical psychological abnormalities. The impact of COVID-19 on demoralization and subsequent interventions and psychological care may become a future research direction.

**Conclusion:**

There has been a significant increase in research in the field of demoralization after 2000. The United States provided the most publications. There is overall active collaboration between authors, countries, and institutions. In future research, more attention will be paid to the effects of COVID-19 on demoralization and intervention care for this psychology.

## Introduction

Demoralization is a constant experience of inability to cope, with feelings of helplessness, hopelessness, meaninglessness, a subjective sense of incompetence, and low self-esteem (Frank, 1974; Clarke and Kissane, 2002). Demoralization may be a precursor to depression, but it does not meet the formal diagnostic criteria for depression (Kissane et al., 2001). Although it is distinct from anhedonia, it often coexists with major depressive disorder (MDD) in individuals experiencing severe psychological distress (Robinson et al., 2015). For a long time in the past, people often sought psychiatric treatment for this, but it was often ignored by psychiatrists and evaded active intervention by the medical world. With the rapid advancement of research in recent years, the condition of demoralization has received increased attention in clinical and psychological settings. It has now been transformed from a single physical and psychological research variable to a diagnostic framework and is included in the Diagnostic and Statistical Manual of Mental Disorders (DSM; Kissane et al., 2017). It has also been listed as a psychological symptom in the latest International Classification of Diseases (ICD-11, Coding: MB22.2; The, 2019) and has received considerable attention from scholars worldwide.

Demoralization is often caused by events or situations, such as psychiatric disorders, chronic illness, or major trauma. As a major stressor, cancer often causes severe psychological changes in patients during its diagnosis and treatment. Psycho-oncology has been given long-standing attention, with its close focus on the psychological distress of cancer patients, carers, and families (Ahmad et al., 2022). Demoralization is one of the essential psychological issues for oncology, and its prevalence is relatively high (Robinson et al., 2015; Chin et al., 2021). In recent years, the concept of demoralization has been extended to more groups such as patients’ family caregivers (Bovero et al., 2022), Parkinson’s disease (Zhu et al., 2021a), organ transplant patients (Tackmann and Dettmer, 2020; Hsu et al., 2021; Rzeszut and Assael, 2021), postpartum women (Arshad et al., 2021), skin diseases (Petito et al., 2020; Sumpton et al., 2020) and inpatients and medical caregivers under the COVID-19 epidemic (Talebi-Azar et al., 2020; Ferrario et al., 2021; Mumoli et al., 2021; Hendrickson et al., 2022).

Jerome Frank first mentioned the concept of demoralization in 1974 (Frank, 1974), and only a few studies were carried out a long time afterwards. It was not until 2002 that Clarke DM, an Australian scholar, formally introduced the concept of “demoralization syndrome” (Clarke and Kissane, 2002). Since then, then the symptom has become more widely known, and related research has gradually been conducted. The number of studies has exploded in recent years. Existing studies of demoralization-related research have mainly used qualitative interviews, cross-sectional surveys, and longitudinal follow-up with limited target groups, such as specific diseases, specific target groups, and unique occupations (Wu et al., 2019; Skelton et al., 2020; Philipp et al., 2021; Zhu et al., 2021a). Due to time and financial constraints and the deviation of analysis results from different professionals, the research mentioned above proposal can hardly provide a comprehensive directional hint for research in the field of demoralization. As such, literature research is better suited to macro-assessment and micro-understanding of the details of a particular academic field (Kim and Park, 2021). Bibliometric research is based on the results of existing studies to assess research features, hot spots, and emerging trends in the field (Niu et al., 2021). It has been used in a wide range of disciplines, including medicine. Current bibliometric methods are mainly combined with computer-aided metrological visualization to graph the related content.

Therefore, analyzing and summarizing the current state of research in the field of demoralization through visual knowledge mapping is conducive to clarifying its research hotspots and directions, deepening researchers’ understanding and exploration of the field, and promoting the healthy development of patients’ psychology.

## Methodology

### Data collection

Data were systematically searched from PubMed, Web of Science, the Cochrane Library, and CINAHL Complete, with citation information supplemented by the Scopus database. “demoralization” and “demorali*” were used as retrieval words, and the subject terms and free word domains were searched jointly. Searches were conducted up to 31st December 2021, and conference abstracts, erratum, and various studies inconsistent with the research topic were excluded. Details of the search strategy are presented in Supplementary Table 1. All literature records and reference data were exported together as the basic data of this study.

### Analysis method

After screening and data cleaning of the retrieved literature, a bibliometric analysis was achieved with the help of several tools. GraphPad Prisma8.2.1 software was used to analyze literature publication trends and total annual citations in different years. At the same time, VOSviewer1.6.16 (Leiden University, Leiden, Netherlands) was used for cluster analysis of author cooperative relationship networks and research hotspots (Fu et al., 2019; Gao et al., 2019). Visual network clustering mainly includes three elements: node size, the distance between nodes, and node color. Nodes usually refer to specific entities, such as research focus or different scholars, and their size represents the frequency of occurrence (Liang et al., 2018). The distance between nodes indicates the degree of affinity between two nodes, the line is the co-occurrence relation, and the thickness of the line is the co-occurrence frequency (Chen, 2006; Xie, 2015), and different colors refer to different clustering relationships (Liang et al., 2017). In addition, the Biblioshiny tool, developed by Dr. Massimo Aria in Italy, is a scientific bibliometric visualization tool based on R Studio (Martynov et al., 2020). Biblioshiny encapsulates the core code of Bibliometrix (Aria, 2017), which creates a data analysis framework based on web pages, enabling users to carry out relevant scientific measurement and visual analysis on an intuitive interactive web interface (Zhu et al., 2021b). In this way, we have fully evaluated and analyzed the cloud of hot words cloud, research trends, cited journals, concept maps, publications, and cooperation between countries in related studies of demoralization.

### Ethic review

Bibliometric information data were obtained from major public databases, and the Scopus database supplemented the citation and cited relationships. Such data extraction and visualization did not yet involve direct contact or interaction with humans. Therefore, there are no ethical issues in the research, and approval from the research ethics committee was not required.

## Results

### Number of publications and growth characteristics

**Figure 1:**
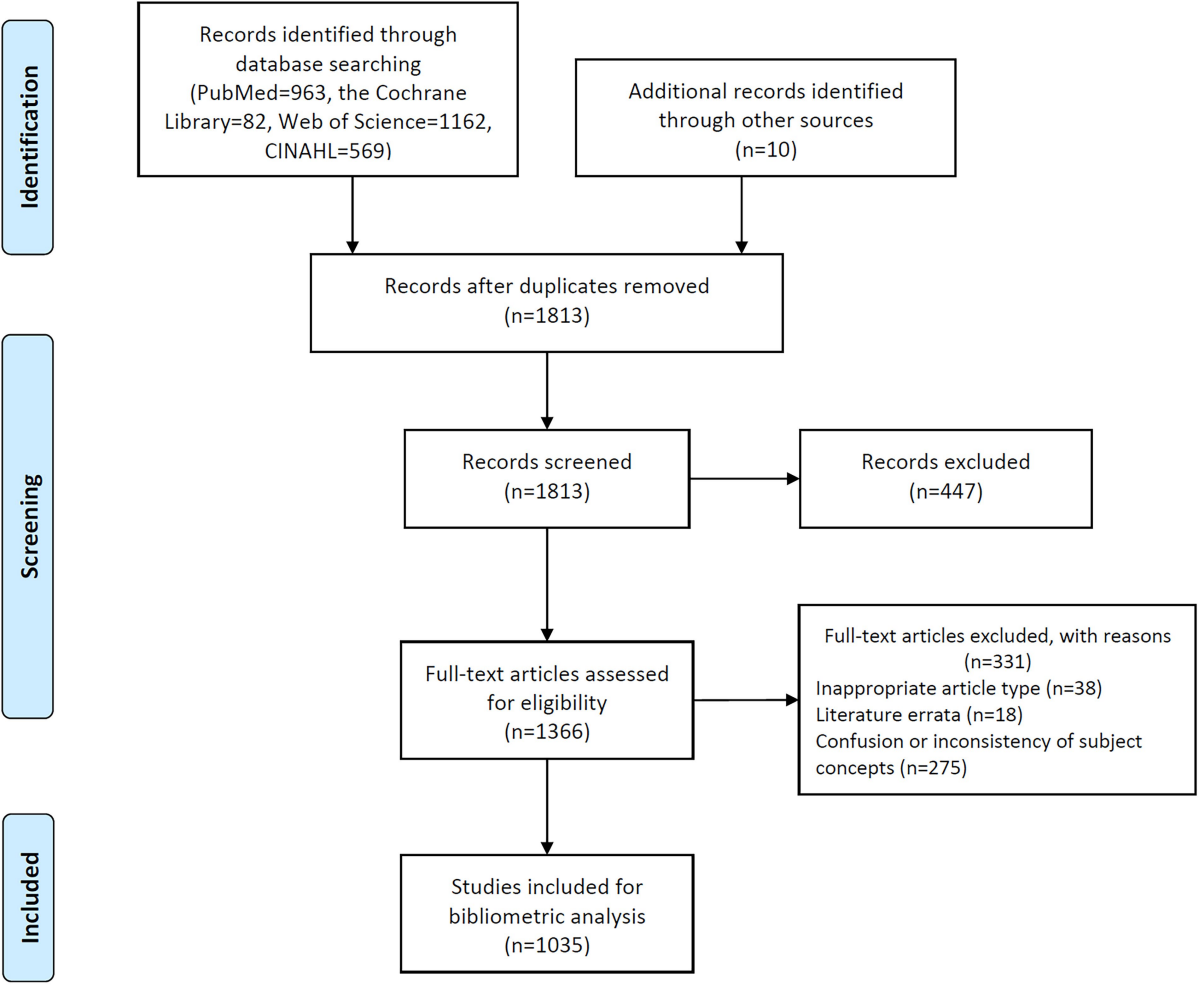
Flow chart of search and screening for related studies of demoralization.

**Figure 2:**
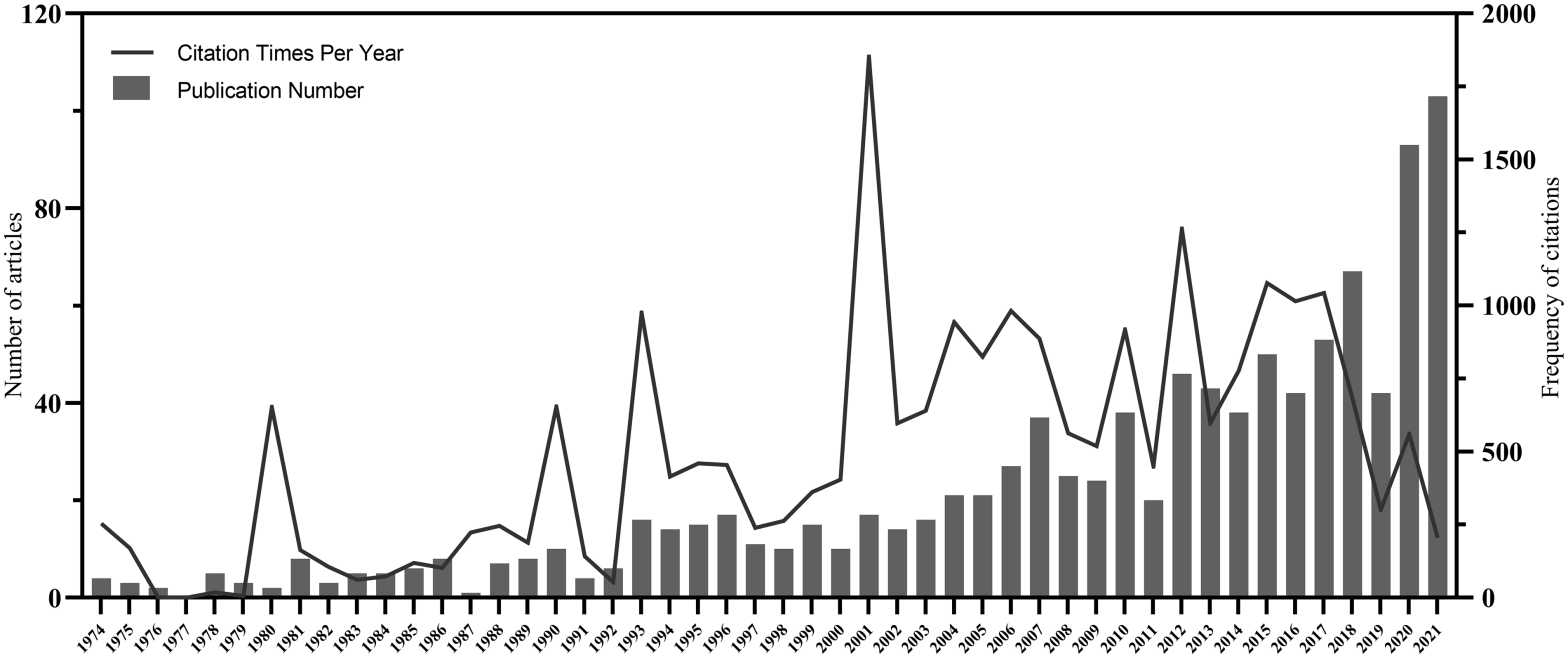
Annual number of publications and citations of demoralization studies.

The initial search retrieved 2,786 documents, and 1,035 were analyzed after de-duplication and careful screening (Figure 1). Figure 2 shows the trend of research publication and annual citation in the field of demoralization since the establishment of databases. The first study on demoralization was published in 1974, and related research was sporadic and tepid until the 21st century. Since the 21st century, there has been a steady increase in the number of articles published in this research area, with the peak of citations in 2001. In the last 2 years, in particular, the enthusiasm for this field seemed to have been further ignited.

### Distribution of source journals

A total of 1,035 studies were published in 657 different journals. Table 1 shows the journals with 10 or more articles on topics related to demoralization. The journals with the most articles were Psycho-Oncology and Psychother Psychosomp, with 25 papers, from the United Kingdom and the Netherlands, respectively. They were followed by Psychosomatics (*n* = 21, 2.03%), Palliat Support Care (*n* = 18, 1.74%) and J Pain Symptom Manag (*n* = 16, 1.55%). More than half of the 12 journals were from the United States, and the rest were from Europe, with the high-volume journal categories being mainly psychiatry and psychology.

**Table 1:** Journals with 10 or more articles on demoralization.

Rank	Journal	Number of articles published (%)	Country	Subject classification*	IF (2021)
1	Psycho-Oncology	25 (2.41)	England	● PSYCHOLOGY, MULTIDISCIPLINARY ● SOCIAL SCIENCES, BIOMEDICAL	3.955
2	Psychother Psychosom	25 (2.41)	Switzerland	PSYCHIATRY	25.617
3	Psychosomatics	21 (2.03)	United States	PSYCHIATRY	3.099
4	Palliat Support Care	18 (1.74)	England	HEALTH POLICY & SERVICES	3.733
5	J Pain Symptom Manag	16 (1.55)	United States	● CLINICAL NEUROLOGY ● HEALTH CARE SCIENCES & SERVICES ● MEDICINE, GENERAL & INTERNAL	5.576
6	J Nerv Ment Dis	14 (1.35)	United States	PSYCHIATRY	1.899
7	Support Care Cancer	14 (1.35)	United States	• ONCOLOGY • HEALTH CARE SCIENCES & SERVICES • REHABILITATION	3.359
8	Compr Psychiat	11 (1.06)	United States	PSYCHIATRY	7.211
9	Psychol Assessment	11 (1.06)	United States	PSYCHOLOGY, CLINICAL	6.083
10	Eur J Psychiat	10 (0.97)	Spain	PSYCHIATRY	1.288
11	J Affect Disorders	10 (0.97)	Netherlands	PSYCHIATRY	6.533
12	J Pers Assess	10 (0.97)	United States	• PSYCHOLOGY, CLINICAL • PSYCHOLOGY, SOCIAL	3.720

*Based on Social Sciences Citation Index (SSCI).

### Highly cited articles

Highly cited articles on a research topic often explain its important status and breakthroughs. Table 2 shows the top 10 cited studies in the field of demoralization. Dohrenwend BP’s 1980 publication, ‘Nonspecific Psychological Distress and Other Dimensions of Psychopathology’ (Dohrenwend et al., 1980), received the most frequent citation with 653 times. Also cited more than 600 times was the social phenomenology study conducted by Cattell V in 2001 (Cattell, 2001). The remaining highly cited articles were all cited between 200 and 500 times.

**Table 2:** Top 10 cited studies.

Rank	Title	Year	Journal	TCs
1	Nonspecific psychological distress and other dimensions of psychopathology Measures for use in the general population (Dohrenwend et al., 1980)	1980	Arch Gen Psychiatry	653
2	Poor people, poor places, and poor health: the mediating role of social networks and social capital (Cattell, 2001)	2001	Soc Sci Med	647
3	Differential diagnosis and classification of apathy (Marin, 1990)	1990	Am J Psychiatry	457
4	Rapid and sustained symptom reduction following psilocybin treatment for anxiety and depression in patients with life-threatening cancer: a randomized controlled trial (Ross et al., 2016)	2016	J Psychopharmacol	443
5	Demoralization: its phenomenology and importance (Clarke and Kissane, 2002)	2002	Aust New Zealand J Psychiatry	328
6	Demoralization syndrome -- a relevant psychiatric diagnosis for palliative care (Kissane et al., 2001)	2001	J Palliative Care	319
7	Depression, demoralization and control over psychotic illness: a comparison of depressed and non-depressed patients with a chronic psychosis (Birchwood et al., 1993)	1993	Psychol Med	308
8	Psychotherapy: the restoration of morale (Frank, 1974)	1974	Am J Psychiatry	232
9	Developmental issues and their resolution for gay and lesbian adolescents (Hetrick and Martin, 1987)	1987	J Homosex	224
10	The Demoralization Scale: a report of its development and preliminary validation (Kissane et al., 2004)	2004	J Palliative Care	215

### Active countries/regions, institutions, and associated researchers

**Figure 3:**
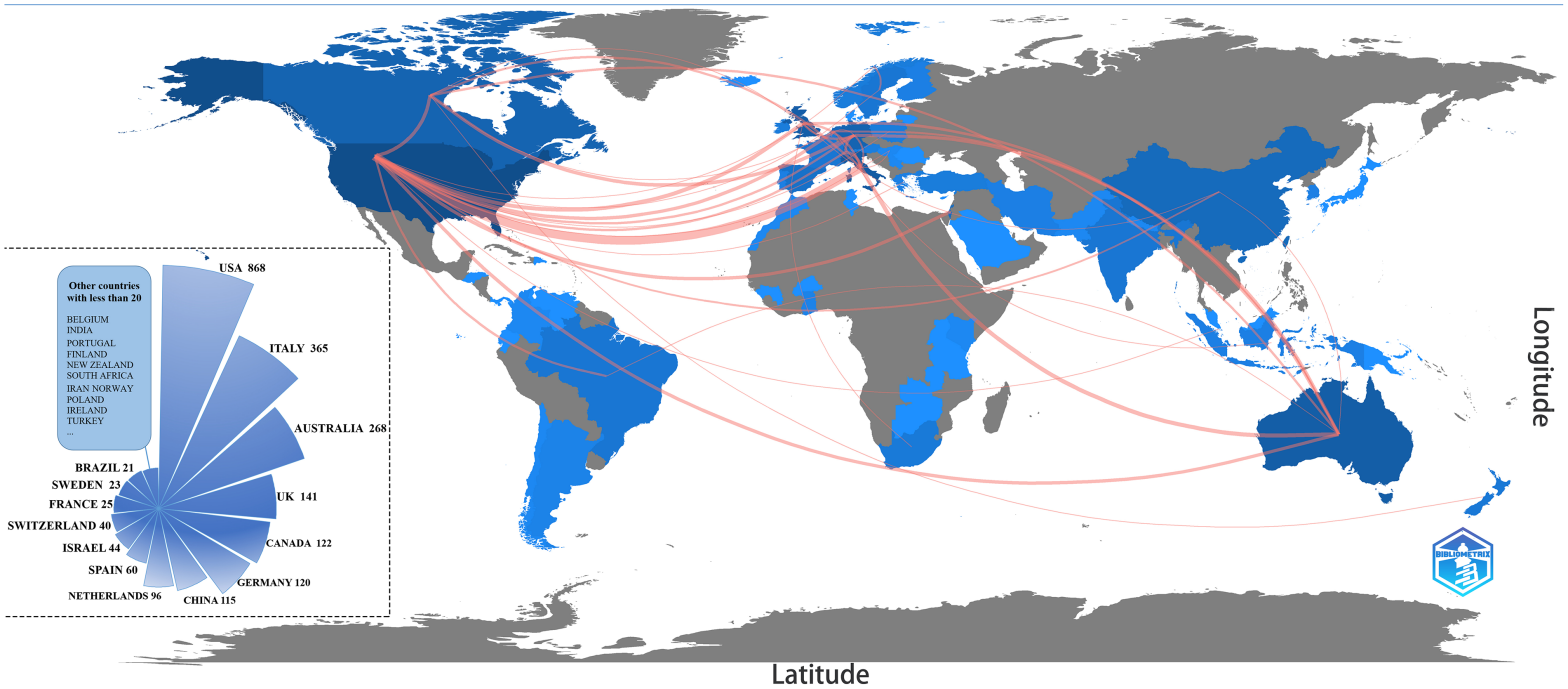
Global publication level and cooperation network.

Figuring out the number of publications and collaborations between different countries can help to quickly identify which countries have made significant contributions to the exploration of demoralization research. According to SCIE, 64 countries were involved in publishing in the field of demoralization. Figure 3 shows the number of publications worldwide and cooperation between countries, with darker colors denoting more publications, and the lines between countries indicating collaborative networks. The majority of research articles were published in North America, Europe, Asia, and Australia, with the United States dominating (34.05%), followed by Italy (14.3%), Australia (10.5%), and the United Kingdom (5.5%) and Canada (4.8%). More than 50 countries, including Belgium, India, Portugal, and Finland, have participated in demoralization studies, but none have published more than 20 articles. Regarding the sparseness of the connections, the United States, as the leading country in research publications, has engaged in close and multiple collaborations with Europe and other countries around the world.

**Figure 4:**
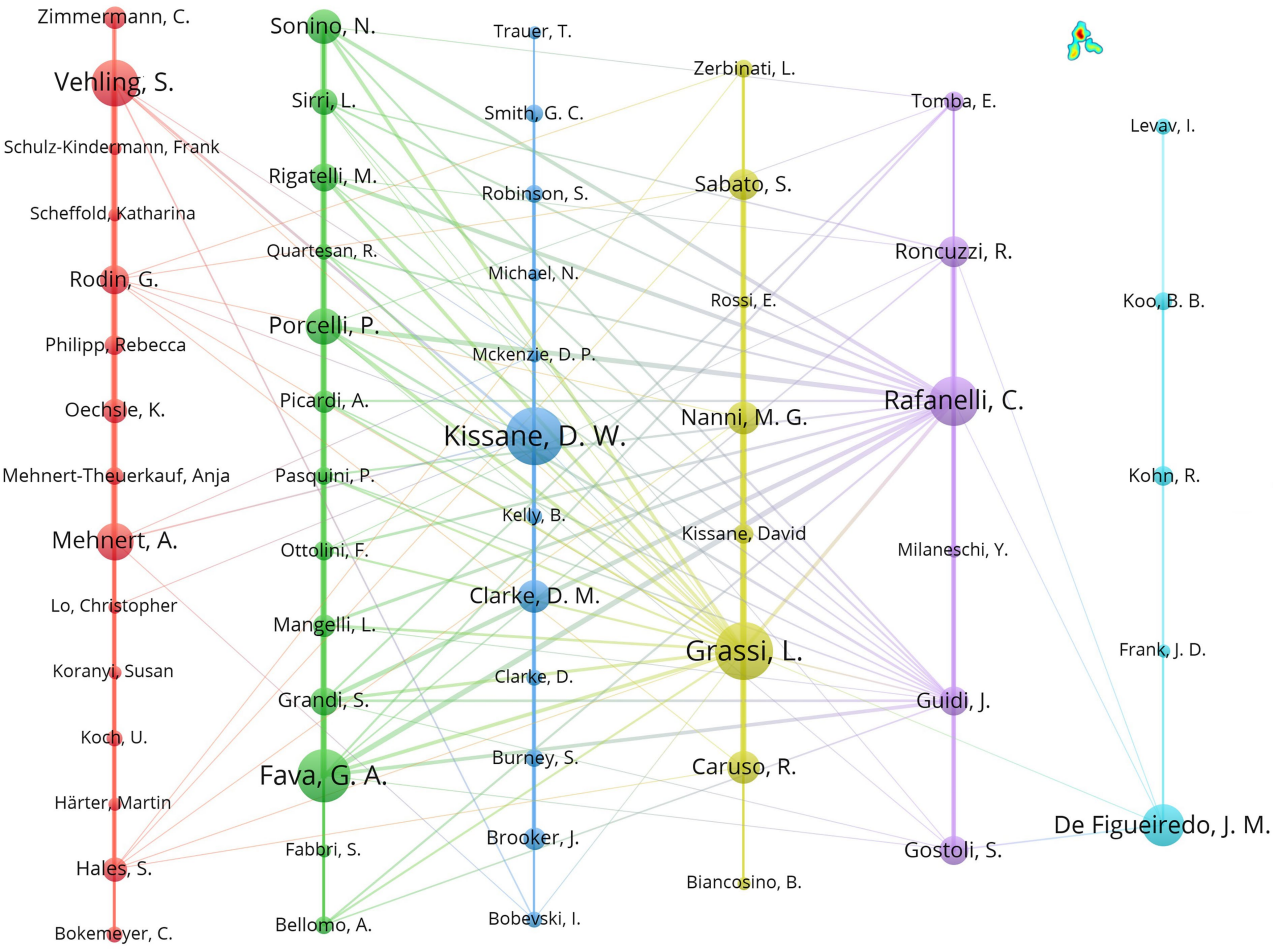
Author collaboration network.

Author visualization was used to analyze the collaboration patterns of authors posting in the field of demoralization. Prolific authors were calculated using fractional counting, with the type and unit of analysis being co-author and author, respectively. After finding and screening for authors of the same name in different abbreviation formats, a posting threshold of 4 was set to facilitate the identification of well-known authors who had published research papers in the field. The results showed that a total of 59 authors were eligible. Figure 4 presents the collaborative network relationships between and among the high publication authors, forming six different collaborative groups. The authors with 15 or more publications, their institutions, and their countries are summarized in Table 3. Grassi L and Kissane DW posted 31 articles and tied for first place. More than half of the nine authors were from Italy, two were from Germany, and the remaining two were from Australia and the United States. They were also central figures in the collaborative groups shown in Figure 4. Apart from Rafanelli C and Fava GA, the University of Bologna scholars and the other highly published scholars were all from different institutions.

**Table 3:** Author and institution information of more than 15 publications.

Rank	Researcher	Number of articles published	Institution	Country
1	Grassi L	31	• University of Ferrara • Anna University Hospital and Health Authority	Italy
2	Kissane DW	31	• Monash University • Cabrini Monash Psycho-oncology, Cabrini Health	Australia
3	Fava GA	28	University of Bologna	Italy
4	Rafanelli C	25	University of Bologna	Italy
5	Vehling S	23	University Medical Center Hamburg-Eppendorf	Germany
6	De Figueiredo JM	20	Yale University School of Medicine	United States
7	Mehnert A	17	University Medical Center Leipzig	Germany
8	Porcelli P	16	IRCCS De Bellis Hospital	Italy
9	Sonino N	15	University of Padova	Italy

### Research hotspot and clustering direction

**Figure 5:**
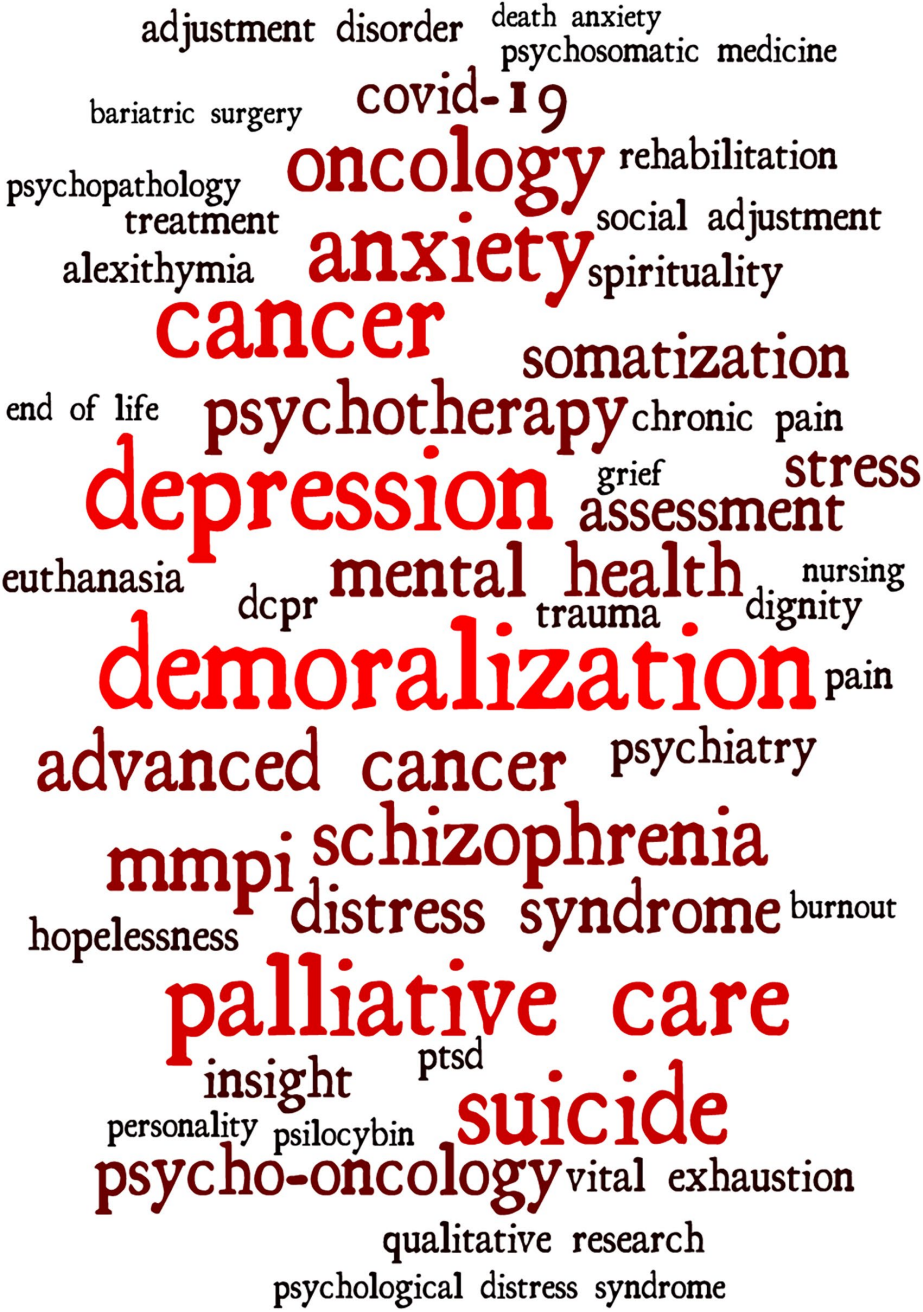
Research hot word cloud map.

**Figure 6:**
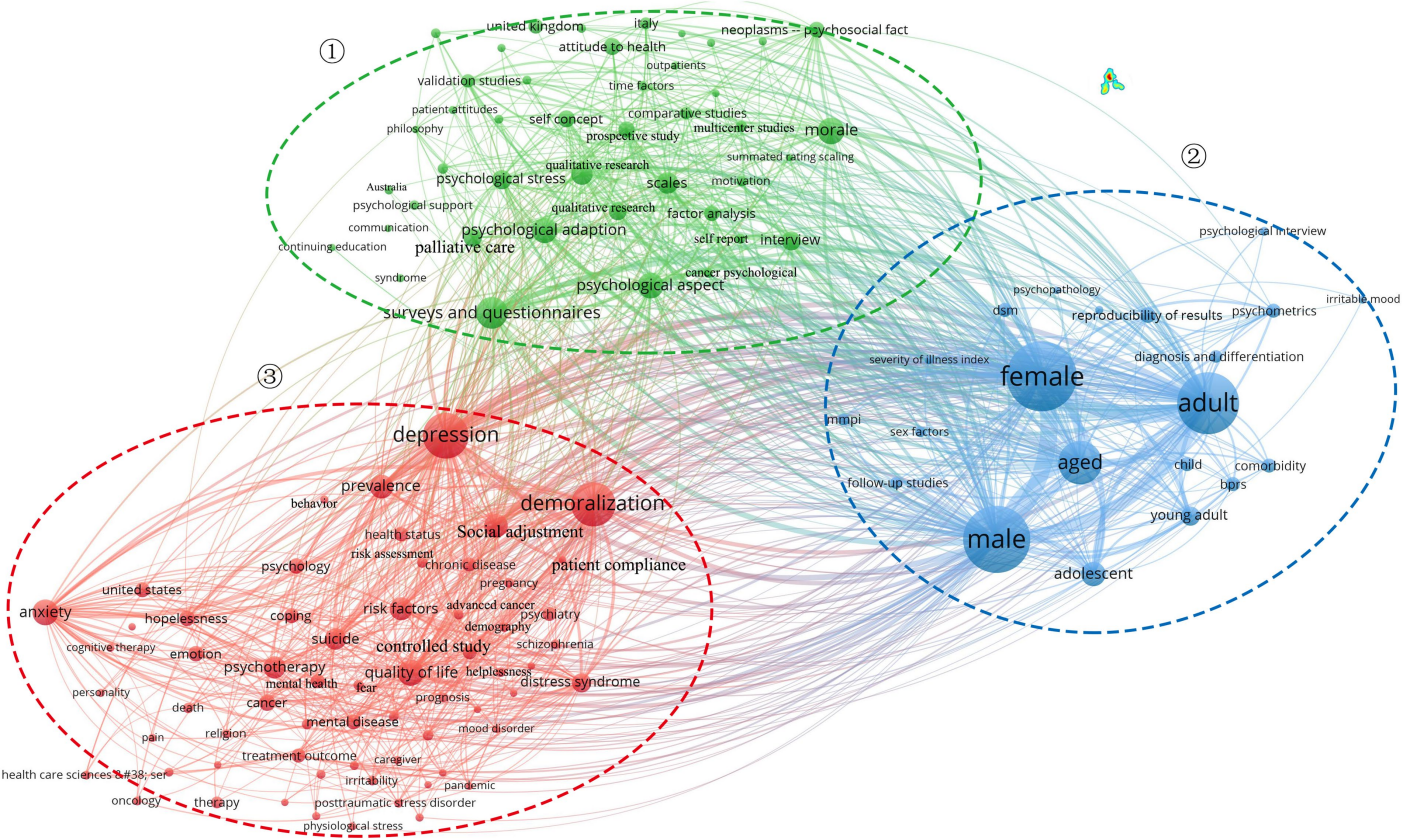
Research hotspot direction and clustering.

Visualized presentation of subject content lines is often a valuable way of assessing research direction on a hot topic. Based on keyword frequency (Top 50), a hot word cloud map in the field of demoralization was created (Figure 5). Demoralization, depression, anxiety, cancer, suicide, schizophrenia, COVID-19, psycho-oncology, and MMPI all had high frequencies of occurrence. Figure 6 shows the clustering direction of the main hot topics in the current study on demoralization. The 127 keywords with 10 or more occurrences were grouped into three categories, illustrating the three main research directions. The first cluster contained 43 keywords, including surveys and questionnaires, semi-structured interview, clinical assessment tools, scales, and qualitative research, concentrating on the assessment tools and multiple study design approaches used in different countries to understand patients’ specific psychological states. The second cluster consisted of 21 keywords covering female, male, psychometrics, comorbidity, aged, and severity of illness index, mainly concerning the distribution of demoralization among different characteristics and the degree of associated psychological complications and their impact. The third group was the largest and contained 63 keywords, including demoralization, anxiety, depression, schizophrenia, advanced cancer, COVID-19, pregnancies, caregiver and health care personnel, which focused on the differentiation and links between demoralization and other psychological disturbances, the psychology of demoralization in various disease contexts and the distribution of this mental illness in different role groups.

### The relationship between hotspot development and trend

**Figure 7:**
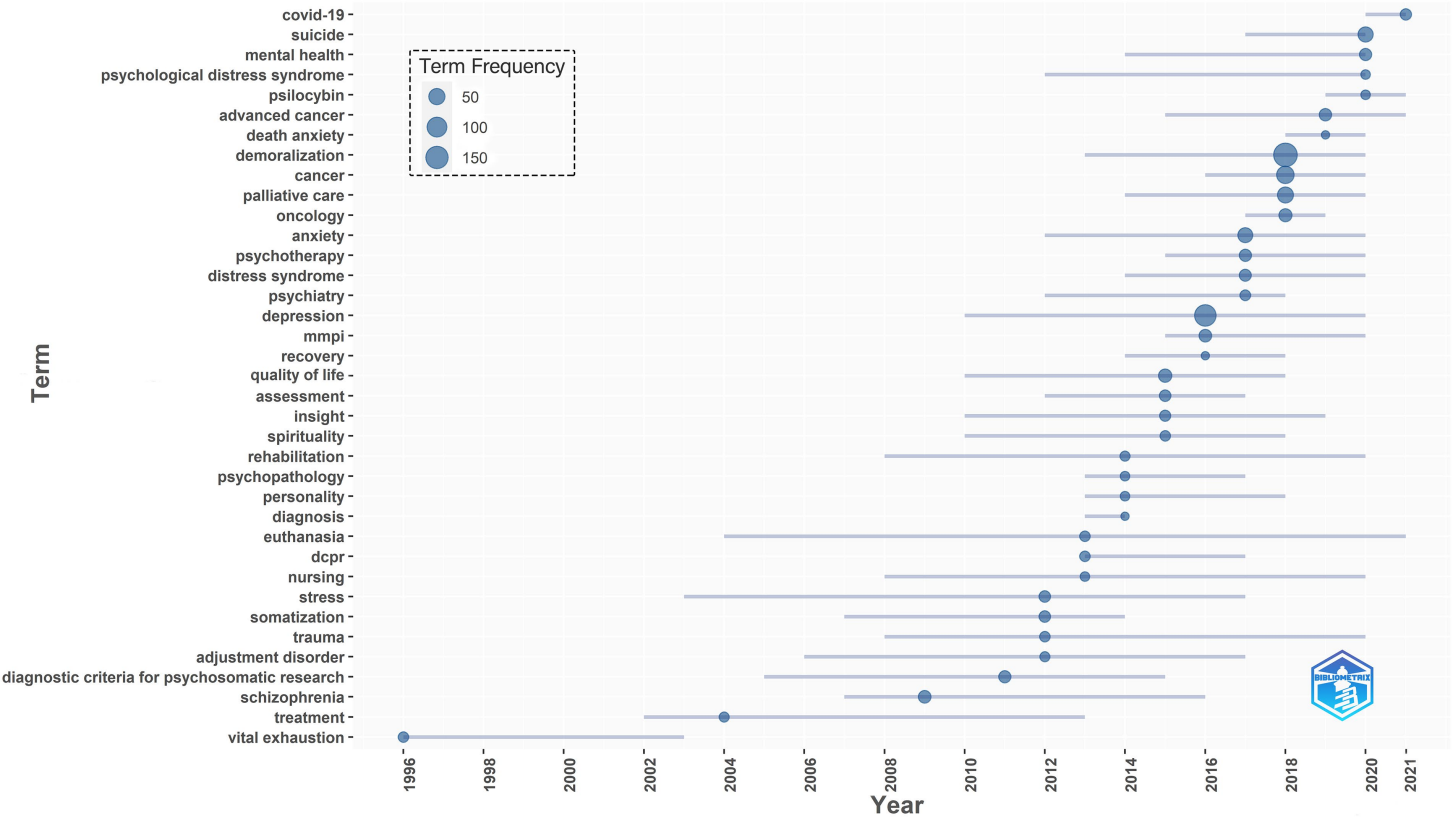
Research hot spots for annual outbreak trends.

Combining hotspots with their year of appearance can better reflect the temporal trend and tendencies of hotspot focus. Figure 7 shows the hot keywords that exploded over the years. The size of the circle reflects the frequency of the keyword outbreak, while the span of the horizontal line refers to the time range of the high frequency of hot spots.

In recent years, the new focus keywords mainly include COVID-19, suicide, psilocybin, advanced cancer, death anxiety, psychotherapy, and MMPI. Moreover, mental health, psychological distress syndrome, palliative care, anxiety, depression, and rehabilitation are the contents that have been paid more attention to for a long time. Since 2004, euthanasia has been discussed as one of the most popular and longest-lasting topics. The topics of vital failure, treatment, schizophrenia, and somatization disorders in demoralization are fading from the limelight.

**Figure 8:**
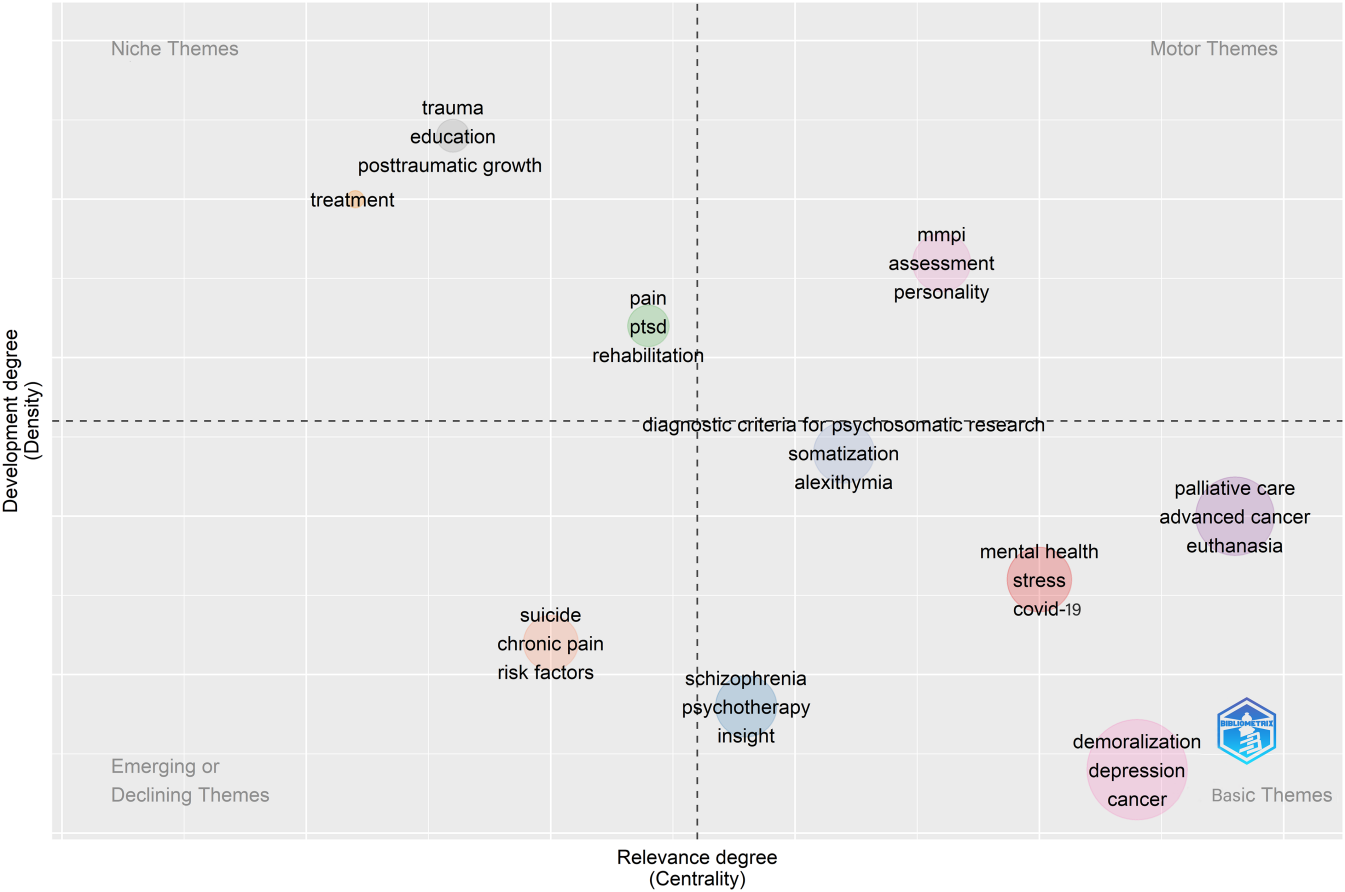
Topic map of research hotspots.

With the help of the biblioshiny tool, a thematic map based on demoralization research was created. The horizontal axis in Figure 8 represents centrality; the vertical axis indicates density. The four quadrants represent motor themes, niche themes, emerging or declining themes, and basic themes. The motor theme included the three main concepts of MMPI (The Minnesota Multiphasic Personality Inventory), assessment, and personality. The issues of trauma, post-traumatic stress disorder (PTSD), and post-traumatic growth in patients (Quadrant 2) were still well evolved but were not as important as other topics in this field. The topics of suicide, chronic pain, and risk factors (Quadrant 3) were among those that have been around for a short time and will soon disappear. The criteria for assessing demoralization, its association and distinction from various types of psychiatric disorders, and the typical phenomenon of demoralization in cancer patients (Quadrant 4) have often been far-reaching as fundamental topics for research in this field. They have provided a solid foundation for the development of the field.

## Discussion

This study conducted a comprehensive bibliometric analysis of the field of demoralization based on multiple databases. This analysis identified research publication trends, distribution of high publication journals, highly cited studies, high-producing authors and national collaborations, and research hotspot clustering and trends.

A total of 1,035 studies were retrieved, spanning 48 years. The average annual number of publications was about 21.6. In the last decade or so, the research volume has significantly exceeded this figure. It may be a result of the ever-growing prevalence of physical and psychological adjustment disorders in the clinical setting. Therefore healthcare professionals were increasingly concerned about the psychological maladies that occurred in patients, of which demoralization was quite common (Grassi et al., 2007). Kissane DW and Clarke DM, scholars from Australia, first explained the concept of “demoralization syndrome” systematically and comprehensively in the early 21st century (Kissane et al., 2001; Clarke and Kissane, 2002). Its phenomenology and importance were clarified and distinguished from other mental problems. Therefore, at that time, the total citation volume of related studies reached its peak and then showed a high state of citation concern. Furthermore, the number of articles also increased year by year. The phenomenal growth of the literature in the last 2 years may be related to the fact that the frequent occurrence of psychological problems of demoralization in the context of COVID-19 and its overall appearance not only in the patient population but also among caregivers and health care workers, has attracted widespread attention (Menon et al., 2021; Vitale et al., 2021; White et al., 2021).

The journals on this topic were relatively scattered, with few high-volume journals. The top 12 journals published nearly 20% of the studies, and the vast majority of journals only published 1–2 relevant studies. However, it is encouraging that high-impact journals in specialized fields such as Psychother Psychosom have preferred this topic.

The top cited studies have all contributed to the progress and breakthroughs in the field of demoralization. An analysis of the top 10 cited studies revealed that there were several significant historical events in the progress of the field of demoralization, involving the conceptualization, the differentiation from other psychiatric symptoms, the investigation, and exploration of potential dysfunctional psychology in people with different backgrounds, the development and application of the standardized demoralization scale (DS-I), and the research based on the exploration of the psychology of demoralization. Frank JD first proposed the concept of demoralization in 1974 (Frank, 1974), which pioneered research in this field. The Psychiatric Epidemiology Research Interview (PERI), developed by Dohrenwend BP in 1980 through a study of 200 adults of different genders, classes, and ethnicities in New York City, made Frank’s first concept and signs of psychopathology tangible and visual in the context of psychopathology (Dohrenwend et al., 1980). It also distinguished it from other non-specific psychological disturbances and contributed to subsequent scholarly understanding of the concept. A study by Hetrick ES in 1987 (Hetrick and Martin, 1987) found that domestic violence, emotional and educational stress, and sexual abuse in adolescents with homosexual orientation, if not adequately addressed, were also associated with anxiety, depression, and demoralization in adulthood. Marin (1990) later further distinguished ‘apathy’ from demoralization as an increasingly sophisticated psychological phenomenon, distinct from other psychiatric signs. Birchwood et al. (1993) also further dissected the similarities and differences between depression and demoralization and considered the control of these psychiatric symptoms. Later, in the 21st century, Cattell V’s research (Cattell, 2001) also found for the first time that the emergence of community characteristics and the perception of inequality could also be the source of demoralization, confirming the prevalence of demoralization among the general population. Subsequently, Kissane et al. (2001) and Clarke and Kissane (2002) systematically explained and defined the concept of demoralization and ‘demoralization syndrome’ for the first time, which also contributed to the explosion of citations to the peak of the literature in the early 2000s, paving the way for subsequent research in the field and furthering the extension and development of the concept of demoralization. Soon afterward, in 2004, Kissane et al. (2004) designed and initially validated the demoralization scale (DS-I) based on the five dimensions of loss of meaning, dysphoria, disheartenment, helplessness, and sense of failure in psycho-oncology and palliative care patients. Creating a concrete assessment framework has also enhanced conceptual understanding and attracted the attention of scholars worldwide, allowing demoralization to be accurately identified from an abstract concept through specific items. For a long time afterward, people have been exploring the determination and recovery of demoralization. In 2016, Ross et al. (2016) attempted a randomized controlled trial and found that psilocybin treatment significantly improved anxiety and depression in advanced cancer patients while significantly reducing patient demoralization and despair.

Among the 64 countries involved in demoralization-related research, the level of focus and depth of research varied widely. The United States was involved in more than one-third of the publications in this research area and has close and multiple collaborations with countries worldwide. However, only one scholar from the United States was included in the distribution of highly productive authors (*n* ≥ 15), while more than half of the scholars were from Italy. Research in the United States is generally more mature, but the most dedicated scholars in the field were still primarily located in Europe and Australia. It was also found that the collaborative author groups were tightly connected internally. In contrast, the collaboration between different groups relied only on the core scholars to sustain them, which needed to be strengthened in the future.

In recent years, the phenomenon of demoralization has come to receive increasing attention. As a specific psychological state, it has often been used to correlate with or differentially diagnose other psychiatric symptoms such as schizophrenia, anxiety, hopelessness, depression, grief, pain, stress, psychological distress syndrome, alexithymia, etc. (Nanni et al., 2018; Tang et al., 2020). Demoralization occurring at the end of life or in patients with advanced cancer tends to receive more attention (Kissane et al., 2017). The main hotspots that should be paid great attention to by caregivers are somatization disorder, adjustment disorder, alexithymia, vital exhaustion, and suicide. In addition, social resilience, palliative care, and specific coping in the context of COVID-19 were often noted in this group of patients (Mumoli et al., 2021).

The results of the thematic cluster analysis revealed that current research in the field of demoralization focused on three different directions. The first direction concentrated on multidimensional and multi-instrumental approaches to assessing demoralization. Diagnosing and assessing demoralization is a prerequisite for considering treatment and targeted interventions. The main tools available are the Subjective Incompetence Scale (SIS; Cockram et al., 2009), the Diagnostic Criteria for Psychosomatic Research (DCPR; Porcelli et al., 2010), the Demoralization Scale (DS; Kissane et al., 2004) and its updated version, the DS-II (Robinson et al., 2016), and a simplified version, the Short Demoralization Scale (SDS; Galiana et al., 2017). Cluster II has mainly explored the occurrence and likelihood of demoralization under different demographic and illness characteristics. But most studies were scattered and have not yet been assessed through evidence-based systematic integration, which may be the next step. The third direction of research focused on the links and distinctions between demoralization and various abnormal clinical psychological problems. In a complex clinical setting, especially with the global epidemic of COVID-19, psychological problems in health care workers are becoming more apparent, and demoralization is a very common part of this (Talebi-Azar et al., 2020; Dean, 2021). In addition to the feelings of despair, meaninglessness, and subjective incompetence that demoralization can bring to individuals, it often seriously impacts the collapse of personal values (Gabel, 2013). The occurrence of demoralization can have a serious negative impact on the running of clinical practice, resulting in higher staff turn-over rates (Santoro, 2011) and reduced job satisfaction, adding to the further breakdown of clinical workflow under high levels of stress (Elder et al., 2020).

The annual hot outbreak trend also illustrates the impact of COVID-19 in driving psychological distress in individuals. Given the current state of the global epidemic, the psychological aspects of demoralization under this perspective will remain a topic of discussion for a long time. Under the haze of demoralization, psychological distress syndrome, death anxiety, and suicide risk of advanced cancer patients have also received significant attention in recent years. As the concept of demoralization becomes more clearly understood, and in contrast to the previous cross-sectional evaluations that focused only on assessing the patient’s psychological status, more exploration and attention has been given in recent years to how this abnormal psychology can be alleviated or cured through pharmacological or psychological care modalities.

The thematic map also plays an overall role in evaluating the topic content in the field of demoralization. MMPI scale was often used to assess personality and psychopathology and is now widely used in clinical and forensic medicine (Wright et al., 2016; Waugh et al., 2021). Its subsequent update, the MMPI-2 Restructured Form (-RF) Spectrum of Personality Disorders Scale, has added a full assessment of demoralization behavior (Sellbom, 2019), making it an essential tool for assessing personality traits for such symptoms and was also well developed. Topics such as trauma, post-traumatic stress disorder, post-traumatic growth, and suicidal chronic pain under the influence of demoralization may soon fade from the research landscape. The typicality of psychological disorders of demoralization in cancer patients was the first to be identified and assessed (Clarke and Kissane, 2002). It was an influential cornerstone of the ongoing exploration of this field and the basis for developing the DS-I (Kissane et al., 2004). From there, more diverse groups of demoralization were only gradually identified and explored.

### Strengths and limitations

To the best of our knowledge, this is the first study to conduct a bibliometric review of demoralization. We used three visualization tools to analyze posting trends, scholar collaboration, hotspot clustering, and trend analysis to demonstrate the current status and future trends in the field of demoralization. However, our study also has some limitations. Although we did not restrict the language type, almost all included papers were published in English, which may result in language bias. Due to the large number of authors and keywords in this study, potential omissions may still occur, although we made adjustments and normalizations during the analysis.

## Conclusion

There is a general growth trend in research in the field of demoralization. Most of its related articles are published in psychology series. However, few journals can focus on the field of demoralization, with most articles published in Psycho-Oncology and Psychother Psychosom. The United States, Italy, and Australia were the three countries that have contributed the most research to demoralization research. Although U.S. scholars were active in demoralization and have published many articles, only a few scholars have become leaders, and the collaboration between different teams also needs to be strengthened. Currently, the research focuses mainly on the evaluation, identification, and influence of different backgrounds on demoralization. In the context of the COVID-19 epidemic, the psychology of demoralization, and its intervention and psychotherapy may become a major direction for future research.

## Author contributions

All the authors conceived the work. QZ and LX: literature search, screening, and inclusion. HL, ML, and JX: statistical analysis and data visualization. QZ and XL: manuscript writing, review, and editing. All authors contributed to the article and approved the submitted version.

## Conflict of interest

The authors declare that the research was conducted in the absence of any commercial or financial relationships that could be construed as a potential conflict of interest.

## Publisher’s note

All claims expressed in this article are solely those of the authors and do not necessarily represent those of their affiliated organizations, or those of the publisher, the editors and the reviewers. Any product that may be evaluated in this article, or claim that may be made by its manufacturer, is not guaranteed or endorsed by the publisher.
