# Supplementary material for: Demoralization: Where it stands-and where we can take it: A bibliometric analysis
Source: Front Psychol. 2022 Oct 20;13:1016601. doi: 10.3389/fpsyg.2022.1016601 (PMC9706393; doi:10.3389/fpsyg.2022.1016601)

Supplementary Material

**Supplementary Table 1**. Search strategy (PubMed).

| **Databases** | **Retrieval strategy** | | **Result** |
| --- | --- | --- | --- |
| PubMed | #1 | "Demoralization"[MeSH Terms] OR "demorali*"[Title/Abstract] | 1,002 |
|  | #2 | **("Demoralization" [Mesh]) OR (demorali*[Title/Abstract])** Filters: **from 1859 - 2021** | 963 |


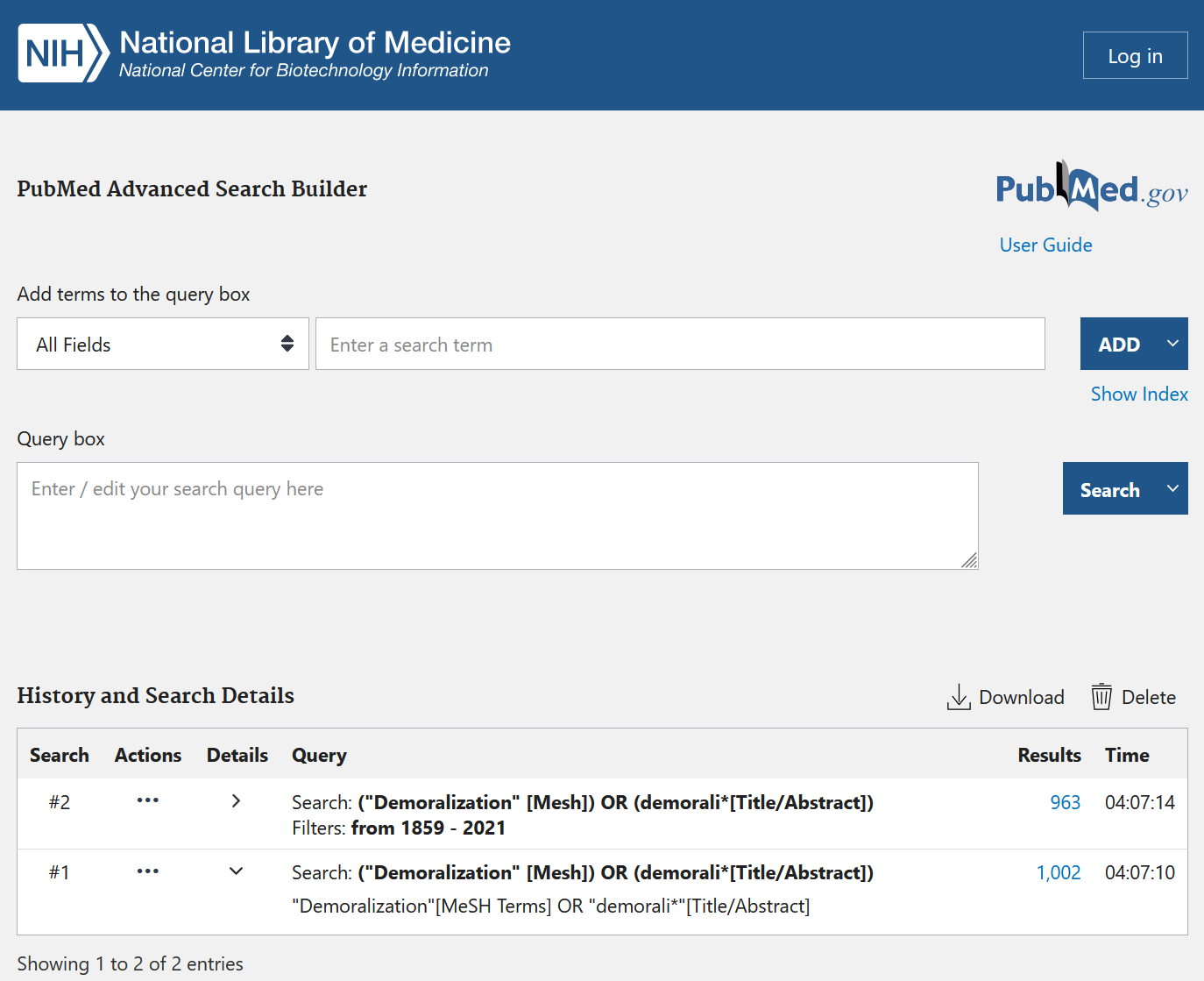

Supplement: Supplementary file 1 [file Table_1.DOCX]
